# Informatics Technology Mimics Ecology: Dense, Mutualistic Collaboration Networks Are Associated with Higher Publication Rates

**DOI:** 10.1371/journal.pone.0030463

**Published:** 2012-01-18

**Authors:** Marco D. Sorani

**Affiliations:** Department of Neurological Surgery, University of California San Francisco, San Francisco, California, United States of America; University of Zaragoza, Spain

## Abstract

Information technology (IT) adoption enables biomedical research. Publications are an accepted measure of research output, and network models can describe the collaborative nature of publication. In particular, ecological networks can serve as analogies for publication and technology adoption. We constructed network models of adoption of bioinformatics programming languages and health IT (HIT) from the literature.

We selected seven programming languages and four types of HIT. We performed PubMed searches to identify publications since 2001. We calculated summary statistics and analyzed spatiotemporal relationships. Then, we assessed ecological models of specialization, cooperativity, competition, evolution, biodiversity, and stability associated with publications.

Adoption of HIT has been variable, while scripting languages have experienced rapid adoption. Hospital systems had the largest HIT research corpus, while Perl had the largest language corpus. Scripting languages represented the largest connected network components. The relationship between edges and nodes was linear, though Bioconductor had more edges than expected and Perl had fewer. Spatiotemporal relationships were weak. Most languages shared a bioinformatics specialization and appeared mutualistic or competitive. HIT specializations varied. Specialization was highest for Bioconductor and radiology systems. Specialization and cooperativity were positively correlated among languages but negatively correlated among HIT. Rates of language evolution were similar. Biodiversity among languages grew in the first half of the decade and stabilized, while diversity among HIT was variable but flat. Compared with publications in 2001, correlation with publications one year later was positive while correlation after ten years was weak and negative.

Adoption of new technologies can be unpredictable. Spatiotemporal relationships facilitate adoption but are not sufficient. As with ecosystems, dense, mutualistic, specialized co-habitation is associated with faster growth. There are rapidly changing trends in external technological and macroeconomic influences. We propose that a better understanding of how technologies are adopted can facilitate their development.

## Introduction

Information technology (IT) innovation and adoption have been significant enablers of recent progress in biomedical research. Biologists were early adopters of computing technology and continue to use it as a primary way of delivering data, tools and knowledge to their communities [1]. For example, recent advances in “next-generation” genomic sequencing have generated vastly larger datasets, so the dissemination of new, automated pipelines for data analysis has been critical in the transition from sequencing innovation to adoption [2]. Early adopters, by necessity, developed custom computer code. More recently, a variety of tools, many developed with open source scripting languages, have become freely available, and the selection of software tools has been driven by the underlying biological questions [3]. Similarly, in clinical practice, physicians are frequently faced with decisions related to adoption of new techniques such as immunization protocols [4] and genetic testing [5], as well as IT such as computer-based documentation software [6] and decision support systems [7], [8]. In contrast to bioinformatics software, some of these types of health information technology (HIT) are decades old, yet while previous obstacles to adoption are being overcome, considerable barriers remain.

Publication in peer-reviewed journals continues to be a well-accepted measure of research output and adoption, and bibliometrics--a quantitative study of textual information--has increasingly used network models to investigate the collaborative nature of research publication. Some network models have been disease-specific in areas like neglected tropical diseases [9], Alzheimer's disease [10], and pulmonary disease [11]. Other examples have developed new methodologies. Douglas et al. developed a web-based tool that extracts relationships from PubMed and maps them to networks [12]; Chen used visualization techniques to reconstruct citation events to examine the temporal growth of a domain [13]; van Eck and Waltman developed a tool that enables multiple graphical representations of bibliometric maps [14]. These studies typically focus on relationships among individuals [15], such as authors, as opposed to relationships among work products, such as research publications or other outputs such as books, patents, videos, and software. These studies observe that simple, local network relationships of nodes and edges assemble into more complex, global features such as node clusters and connected components which suggest robust, large-scale associations. Furthermore, these global features have variable properties such as densities which describe the strength or weakness of certain associations.

Ecological network models can serve as apt analogies for technology adoption. Individual researchers, research institutions, and companies also inhabit “ecosystems” [16]. These are networks of collaborators, competitors, suppliers, and funding organizations. Because researchers, like organisms in a biological ecosystem, ultimately share their fate with the network as a whole, successful researchers often pursue strategies--whether as a “niche player”, a “keystone”, or a dominator--that benefit their ecosystem. Ecological systems can be quantitatively studied, both at the species level using metrics such as specialization to describe individual roles or at the system level using metrics such as stability to describe sets of individuals. There are numerous parallels between natural ecosystems and “systems” of technology adoption. In both cases, it is difficult to study systems in isolation, and it is difficult to perform perturbation experiments due to the lack of adequate controls [17]. There are also differences. External limits described in ecosystems, such as geography, do not appear to inhibit technological and data dispersion (as demonstrated when an online, science-oriented parody of a popular music video was seen over one million times globally in a span of just a few weeks [18]), and society does not appear close to a carrying capacity for technology [19].

In this study, we constructed network models of IT adoption based on the biomedical literature and assessed their ecological properties. Both IT and the literature are vast in scope. Thus, we first focused on two specific areas of IT--programming languages commonly used in bioinformatics research and HIT systems commonly implemented in clinical settings. We further focused on literature published since the first months of 2001 which witnessed seminal publications in bioinformatics related to the initial sequencing of the human genome [20] and a report on the quality of health care in America [21]. We propose that a better understanding of the spatiotemporal characteristics of technology adoption, as well as descriptive and visual models of its topological and ecological network characteristics, can potentially facilitate technological adoption in biomedical research. We found that publications related to open source scripting languages have been rapidly adopted in the last decade, while publications related to commercial languages and HIT grew slowly and variably. Spatiotemporal relationships can facilitate adoption but are not sufficient. As with biological species, dense, mutualistic co-habitation is associated with faster growth.

## Methods

The field of IT is vast in scope. Using the Medical Subject Headings (MeSH) ontology, we selected two well-known areas of “Information Science” [L01] for this study: “Programming Languages” [L01.224.900.780] and “Information Systems” [L01.700.508.300]. Because “Programming Languages” is a terminal branch of the ontology, we used the Google Code Search (http://codesearch.google.com) listing of languages and selected seven: Fortran, JavaScript, Mathematica, Perl, Python, R, and Visual Basic. From the “Information Systems” category in MeSH, we selected four sub-categories: “Clinical Laboratory Information Systems” (LIS) [L01.700.508.300.110], “Hospital Information Systems” (HIS) [L01.700.508.300.408], “Radiology Information Systems” (RIS) [L01.700.508.300.780], and “Reminder Systems” (RS) [L01.700.508.300.790] for further investigation.

Using the four HIT categories and the seven programming languages, we performed PubMed searches of English language articles with abstracts and occurrences of the search term in the title or abstract. The term “Bioconductor” was queried instead of “R” to increase search specificity, and the term “Clinical” was left out of the query for “Laboratory Information Systems”. Programming language searches were limited to references after 2/15/2001 [20]; HIT searches were limited to references after 3/1/2001 [21]. False positives were returned both in the languages selected (e.g., python as a snake, perl as an abbreviation for the peroneus longus muscle in the leg) and in others that were considered but not selected (e.g., ruby as a gemstone, Java as an island, PHP as a gene, and Matlab as a city in Bangladesh), more commonly in older references. We estimated search precision as 0.85 to >0.90 for languages. HIT searches were highly precise (>0.97).

Results were downloaded as MEDLINE files and parsed using custom Python code. We extracted the PubMed ID (PMID), date (DA), place (PL), authors (AU), and journal (TA) fields. The date was truncated to the four-digit year. We calculated summary statistics, including publications per year, cumulative publications over time, the fraction of the decade's publications per year per technology, and compound annual growth rates (CAGR) for publications as measures of adoption rates. We expected correlation between research publications and other modes of publication such as popular books and software code, though we expected less correlation with downstream industrial products such as patents and job listings. We first searched Google Labs' Ngram Viewer [22], a sampling of the entire corpus of published books. We then reviewed current sales data for programming language books [23].We compared publication data with the log transformed counts of search results for the technologies in Google, YouTube, Amazon, the U.S. Patent and Trademark Office, the Library of Congress, Nature Jobs, Craigslist (San Francisco Bay Area), LinkedIn, and Google Code.

We then calculated the geographical distribution of publications to generate statistics and maps, the latter as a means of investigating spatial networks. From the MEDLINE files, we tabulated frequencies of the country of origin for published studies. We color-coded countries according to which technology was most commonly published. Using the frequency data, we generated maps to display the extent to which technology adoption was influenced by geography.

We next generated publication networks. We converted the MEDLINE data into node-edge-node triplets (e.g., PMID1-Author-PMID2) that we imported as undirected graphs using Cytoscape software (http://www.cytoscape.org). Then, we generated attribute files defined by “node  =  type” statements (e.g., “PMID1 =  Perl”). We used these files to visualize and analyze the networks over time and by technology. From this process, we tabulated network topology statistics.

Finally, we applied species- and system-level ecological metrics to the publication data. At the level of individual technologies, we (1) assessed specialization as the fraction of publications per technology occurring in a single journal. We assessed (2) mutualism and competition based on technologies published in similar journals. We then calculated (3) cooperativity as the number of authors per publication, and for languages, we assessed (4) evolution as the temporal progression of time-to-event for version releases [24] (http://cran.cnr.berkeley.edu). At the level of technology families, we calculated (5) biodiversity using -∑p*log(p) as a measure of entropy, where p is the probability that a publication discusses a certain technology. We also estimated (6) stability as the linear fit and Pearson correlation in successive years of publication numbers across technologies. Stability was estimated by comparing the number of publications for each language in 2010 and 2002 versus 2001 to indicate long- and short-term stability. To explore whether past publication frequencies could describe stability and predict future frequencies, we also calculated a Pearson correlation matrix for each technology across the ten years from 2001 to 2010.

## Results

### Technology adoption rates

The literature search identified 2416 total publications. Among HIT, we identified 418 for HIS, 226 for RS, 153 for LIS, and 133 for RIS. Among programming languages, we identified 443 for Perl, 255 for Bioconductor, 244 for Python, 197 for Visual Basic, 166 for Fortran, 95 for Javascript, and 86 for Mathematica. Publication attributes were always reported, except place (PL) which ranged in reporting frequency from 93% (Python) to 99% (four technologies).

**Figure 1:**
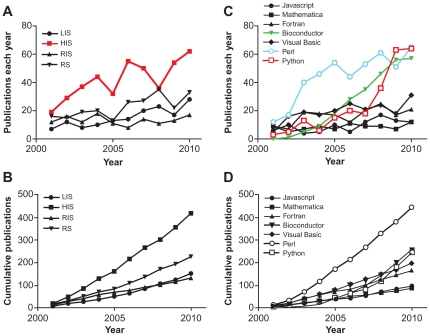
The adoption of technologies over time. Line graphs of the number of PubMed results for each HIT over time, (A) each year and (B) cumulatively. The number of publications on hospital information systems began to grow rapidly in the early part of the decade but has been variable. Overall, there are nearly twice as many HIS publications versus other HIT. (C, D) Corresponding graphs for programming languages. The number of publications using Perl grew rapidly in the early part of the decade and then stabilized, while the number of publications using Bioconductor and Python has grown more recently. Overall, Perl enjoys a nearly two-fold lead in total publications versus other languages.

We first analyzed the frequency of technology publications in the literature over time. Overall, there are substantially more HIS publications versus other HIT. Adoption of HIS appears to be variable over time (Fig. 1A). Cumulatively, there were fewer than 20 total publications for any of the four HIT in 2001, but since then HIS publications has grown to become the greatest presence, with 418 publications in 2010, while RS are second with 226 (Fig. 1B). Among languages, Perl experienced rapid early adoption, while Python and Bioconductor have experienced accelerated adoption more recently (Fig. 1C). There were no more than 12 total publications for any of the seven programming languages in 2001, but since then Perl has grown to have the greatest presence, with 443 publications in 2010, while Bioconductor was second with 255 (Fig. 1D). CAGR ranged from 3% for Mathematica to 40% for Python. No technology in this study experienced decreases.

Because the scientific literature is a specialized corpus, we compared frequency trends for publications of technologies with other broader sources. We observed some divergence between Google Labs' Ngram Viewer and the scientific literature (Fig. S1). For example, among HIT, the frequency of HIS books exceeded that of other HIT, consistent with research publications, but among programming languages, the frequency of JavaScript books exceeded those for Bioconductor. We also observed results that were divergent between Google searches of the Internet and the scientific literature. Occurrences of JavaScript exceeded those of other programming languages, while Visual Basic, Python and Perl showed similar frequencies. Among HIT, RIS were least frequent, while other technologies were similar. One of the biggest recent trends in technology, web development, was down 28% from 2009 to 2010, as measured by book sales--only two areas showed growth: JavaScript and the social web. The categories with the worst performance included Visual Basic. The area showing the most growth among specialized languages was in statistical languages (e.g., R, Mathematica).

Finally, we assessed correlations between frequency counts of technology publications with counts from websites in various socioeconomic domains. Some web sources, such as Amazon (r = 0.04), the Library of Congress (r = −0.08), and the United States Patent and Trademark Office (r = 0.03) were amenable to longitudinal analysis, so we compared web search results with cumulative publication totals. Other web sources, such as Craigslist San Francisco (r = 0.10) and Nature Jobs (r = 0.71) were more amenable to “snapshot” analysis, so we compared web search results with 2010 publication totals. The correlation between Nature Jobs listing counts and 2010 publications was the only one greater than 0.20 (Fig. S2). Still others were compared to both 2010 and cumulative publications, respectively: Google (r = −0.06, 0.01), Google Code (r = −0.18, −0.08), LinkedIn (r = −0.01, 0.00) and YouTube (r = 0.10, 0.04) search results all showed negligible correlation with publication totals.

### Geographic distributions

**Figure 2:**
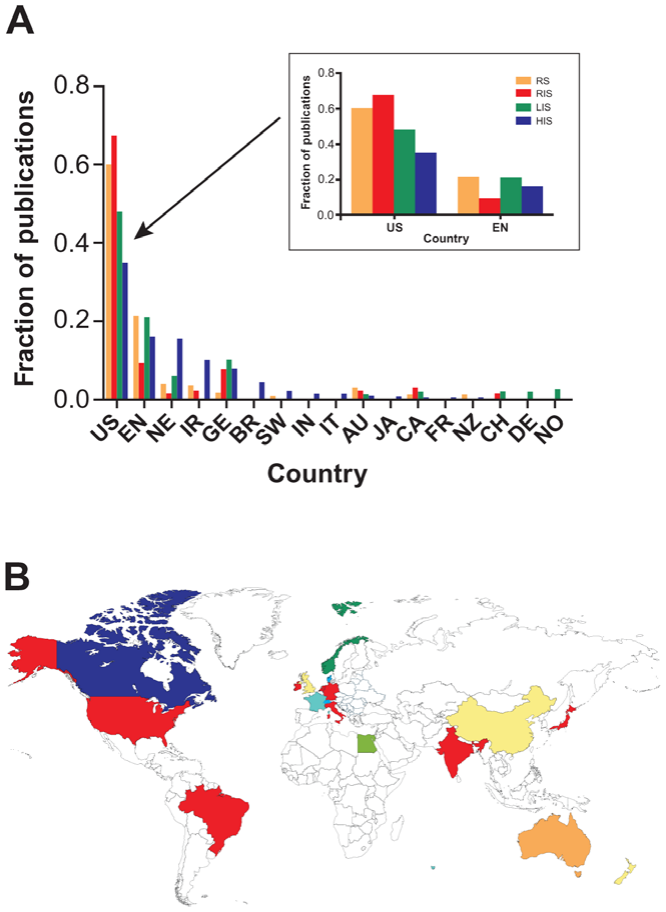
The role of geography in technology adoption. (A) Barchart displays country-of-origin frequencies. Among HIT, the United States publishes more frequently on RIS then does England. (B) World map shows countries of origin for publication. We color-coded countries according to the most commonly published technology. Mathematica, JavaScript, and Fortran did not meet this criteria for any country. Bioconductor is shown in light green, Visual Basic in light blue, Python in aqua, Perl in yellow, RS in orange, RIS in dark blue, LIS in dark green, and HIS in red. The map displays expected clusters in North America, Europe, East Asia, and Asia-Pacific.

We then examined the role of geography in technology adoption. The distribution of publications is similar between programming languages and HIT, with the United States, England, and the Netherlands occupying the top three positions for both families of technology (Fig. 2A). Within technology families, there is considerable variation in the relative publication frequencies. For example, among HIT, the United States publishes more frequently on RIS then does England. Among programming languages, England publishes more frequently on scripting languages, while the US publishes more frequently using commercial products (Fig. S3). Specifically, 75% of Bioconductor publications and 66% of Perl publications originated in England, while 46% of Visual Basic publications and 47% of Mathematica publications originated in the United States. The two countries were nearly equal in JavaScript (44% versus 42%) and Fortran (40% versus 43%) publications.

A world map shows countries of origin for publications of any technology and displays expected clusters in industrialized North America, Europe, East Asia, and Asia-Pacific (Fig. 2B). Mathematica, JavaScript, and Fortran were not the most commonly published technology in any country. Overall, there was little global geographical correlation. For example, among HIT in emerging markets (Fig. S4), China published in radiology and laboratory information systems, while Brazil and India published in hospital information systems. Publication in these areas was more frequent in Northern Europe than Southern Europe.

### Network features

**Figure 3:**
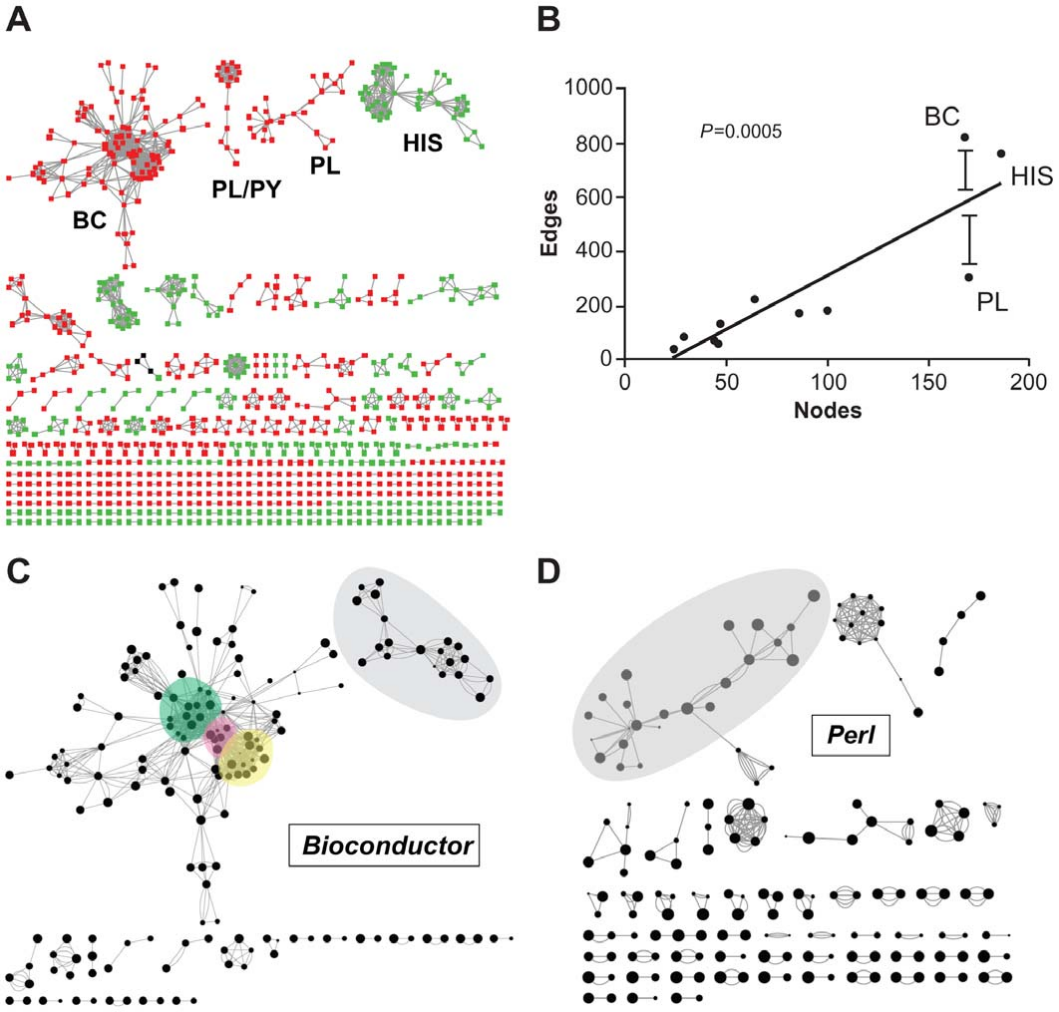
Network characteristics for specific technologies. Published studies are depicted as nodes and common authors as edges. (A) Network colored by technology, languages in red, HIT in green. The largest connected components represent programming languages, specifically Bioconductor, Perl, and Python. The largest component representing HIT is related to HIS. There is only major hybrid component. (B) The relationship between edges and nodes is linear, though Bioconductor has more edges than expected and Perl has fewer. (C, D) Larger nodes are more recent. Nodes of similar time periods tend to be neighbors, though this is less frequently true among more mature languages. Distinct topical clusters are highlighted and described in the text.

We next assessed the temporal and topological characteristics of technology publication networks. Network diagrams of studies as nodes and common authors as edges (Fig. 3A) showed the largest connected components represented scripting languages. The network of HIT was composed of a small number of large connected components, and a large number of components with less than five nodes. The two biggest HIT components represented HIS, and two of the next three biggest components represented LIS. The first component exclusively representing RIS was the 13th largest. Among programming languages, the biggest connected component represented Bioconductor. The next largest, which surprisingly was also the only hybrid component, represented Perl and Python. The next eight largest components represented these three languages. The relationship between edges and nodes was approximately linear, though Bioconductor had more edges than expected and Perl had fewer (Fig. 3B).

Temporal network diagrams were generated of studies published for specific programming languages (Figs. 3C, 3D). Nodes of similar time periods tended to be neighbors, though this was less frequently true among more mature languages. We examined these networks for clusters. The large Bioconductor component is composed of two large and several small clusters (Fig. 3C). The cluster in the lower right represents flow cytometry studies, the bottleneck represents sequencing and network studies, and the cluster in the upper left represents gene expression studies. The second largest connected component represents studies related to SNP microarray analysis, particularly for the Illumina BeadArray platform. The largest connected component in the Perl network reflects the strengths of the language for string manipulation (Fig. 3D). Publications are focused on areas of sequence analysis including interspecies comparisons and comparative genomics, as well as SNP detection, sequence alignment and analysis, and genome annotation.

### Species characteristics: specialization, cooperativity, competition and evolution

Next, we examined the species-level ecological characteristics of different technologies. As previously discussed, based on total publications (Figs. 1C, 1D), some technologies (e.g., Perl and HIS) are dominant species, while others (e.g., Mathematica and RIS) are niche species.

**Figure 4:**
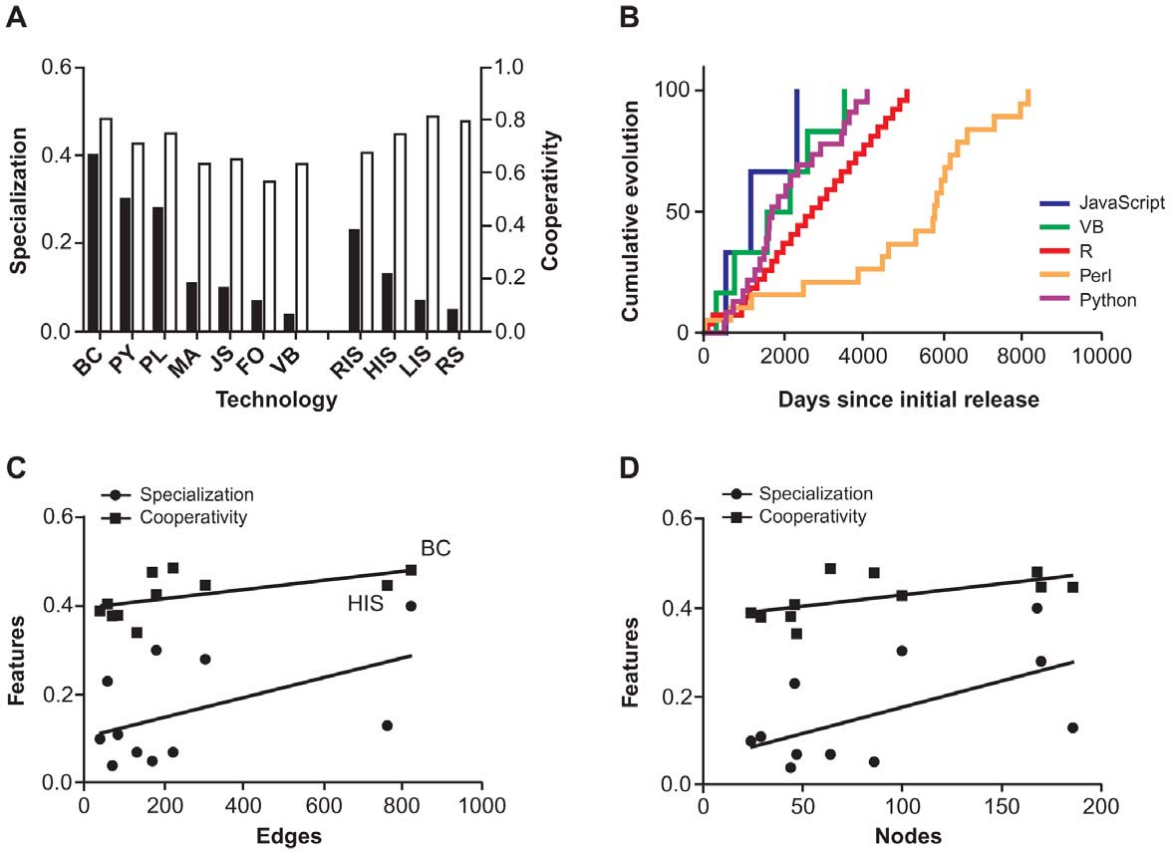
Ecological characteristics of different technologies. (A) Barchart displays specialization measured as the fraction of PubMed results for each technology published in the most common journal, and cooperativity as the number of authors per publication. Among languages, all were specialized in the field of bioinformatics except Fortran. Bioconductor was most specialized, while Visual Basic was least specialized. Among HIT, RIS was most specialized while RS were least specialized. Cooperativity between languages and HIT were comparable. Among languages, specialization and cooperativity were positively correlated, while among HIT they were negatively correlated. (B) Time-to-event curves depict rates of evolution, as measured by time to version releases for languages. Perl's early evolution was slower but then accelerated. Plots of specialization and cooperativity versus (C) network edges and (D) nodes. P-values of linear fits are (C) 0.11 and 0.07, (D) 0.06 and 0.04.

We first assessed specialization as the highest fraction of publications for each technology published in a single journal (Fig. 4A). The fractions ranged from 0.04 for Visual Basic to 0.40 for Bioconductor, a spectrum of greatest generalizability to greatest specialization. The most common journals for six of the seven languages were bioinformatics journals--Fortran publications most often appeared in a computational chemistry journal. Because most of the languages shared a common niche, we consider them to be potentially mutualistic or in competition. Qualitative review of other common journals of publication suggested different secondary specializations, with Python commonly used in neuroinformatics studies, Mathematica in engineering and imaging, JavaScript in medical informatics, and Visual Basic in behavioral research (Fig. S5). On the other hand, the HIT have different and lower degrees of specialization. The fraction of publications ranged from 0.05 for RS to 0.23 for RIS.

Cooperativity among scripting languages and HIT was comparable. Cooperativity ranged from 4.8 for Bioconductor to 3.4 for Fortan, and from 4.9 for LIS to 4.0 for RIS. Older, commercial languages lagged. Interestingly, among languages, specialization and cooperativity were positively correlated (r = 0.93), while among HIT they were negatively correlated (r = −0.98). Finally, for programming languages, we also performed time-to-event analysis of rates of evolution, as measured by time to new version release (Fig. 4B). Across languages, rates of evolution were similar. Perl began as an exception, but around the release of version 5.8 rapidly accelerated its release schedule.

Relationships between cooperativity and specialization versus network node and edge counts were either statistically significant or marginally significant (p = 0.04 and 0.06, 0.07 and 0.11, respectively) (Fig. 4C, 4D). Relationships versus nodes per connected component were not significant (p = 0.87 and 0.41).

### Ecosystem characteristics: biodiversity and stability

**Figure 5:**
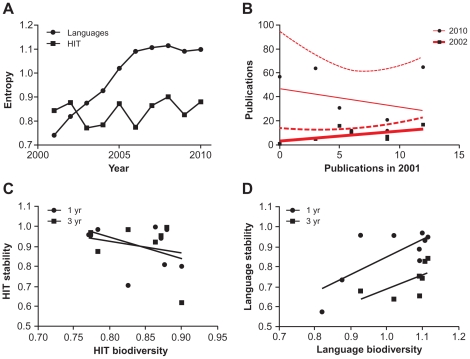
Ecological characteristics of technology families. (A) Biodiversity is estimated using entropy calculations, indicating that diversity among languages grew in the first half of the decade and stabilized, while diversity among HIT has been variable but flat. (B) Stability was estimated by comparing the number of publications for each language in 2010 and 2002 versus 2001 to indicate short- and long-term stability. One-year correlation was positive but not statistically significant; ten-year correlation was negative and weak. Relationships between one-year and three-year stability with biodiversity were (C) negatively correlated for HIT (p = 0.46, 0.38) and (D) positively correlated for programming languages (p = 0.02, p = 0.15).

Biodiversity was estimated for languages and for HIT using entropy calculations (Fig. 5A). Data indicate that diversity among languages grew in the first half of the decade and stabilized, while diversity among HIT has been variable but flat. One-year stability correlation was positive but not statistically significant (r = 0.57, p = 0.18); ten-year correlation was negative and weak (r = −0.26, p = 0.58) (Fig. 5B). We also found that while publication frequencies were correlated with frequencies from previous years (r>0.80), the correlation with data from three years earlier was variable, suggesting that the relative popularity of technologies is unstable and that current states are not predictive of future states, even within a short year time horizon (Fig. S6). We compared one-year and three-year stability with biodiversity for both HIT and programming languages (Fig. 5C, 5D). For HIT, we found non-significant negative correlations (p = 0.46, 0.38). For languages, we found a significant positive relationship between one-year stability and biodiversity (p = 0.02). Three-year stability showed a positive but non-significant trend (p = 0.15). We summarize the relationships among technology, publication, network, and ecosystem attributes and entities in Table 1.

**Table 1:** Mapping of technology, publication, network, and ecosystem attributes and entities.

Technology	Publications	Metric	Networks	Ecology
Implementation	Publication	Count	Node	Organism
User	Author	Cooperativity	Edge	Relationship
Application	Related publications	Density	Cluster	Herd
Release	Date	Stability	Node attribute	Age
Specific Language\System	Low-level MeSH term	Biodiversity, CAGR	Node attribute	Species
Technology type(Language or System)	High-level MeSH term	Count	Node attribute	Genus
Field	Journal	Specialization	Node attribute	Niche

## Discussion

Despite the potential benefits of IT innovation, the adoption of new technologies can be unpredictable and challenging. It has been recognized that adoption and implementation are different phenomena [25], and acquiring a new technology does not necessarily equate to its effective utilization [26]. Furthermore, IT enables but does not guarantee organizational change [27]. Researchers may rely on early adopters to assess new technologies, and early adopter experiences play a role in future adoption patterns. Thus, there is a difference between current users and intended adopters [28], as well as among light, moderate and heavy users [7]. Key barriers to adoption of programming technologies include the organizational learning curve, and barriers to HIT adoption include cost.

In this study, we calculated statistics, constructed network models for analysis and visualization, and described ecological characteristics of biomedical IT adoption, specifically programming languages used in bioinformatics and health IT systems, based on the literature from the last decade. Adoption of HIT has been variable, while scripting languages have experienced rapid adoption. We found that, as with biological species, dense, mutualistic co-habitation is associated with faster growth. We propose that understanding why and how technologies are adopted could aid in their development and lead to scientific and socioeconomic benefit.

### Rapid adoption of scripting languages

The history of programming languages spans more than half a century [24], [29]. Fortran--an acronym for “formula translation” because it was designed to allow translation of mathematical formulas into code--was released in 1954 and was the first high-level language using the first compiler ever developed. It was followed by Lisp (1958) and COBOL (1959). C first appeared in 1971 as a low-level systems language and was followed by Objective C and C++ in 1983. Then, Perl was released in 1987, and Python followed in 1991. These newer scripting languages simplified programming by eliminating the need to manage low-level details such as memory management, allowing programmers to focus on application logic and rapid prototyping. They can also be “bridged” to other languages. For example, the R language is useful for statistical computing, and the RPy library provides an interface between Python and R [30].

We observed that Perl experienced rapid early adoption, while Python and Bioconductor have experienced accelerated adoption more recently. The fraction of publications per year has grown most rapidly for Python and most slowly for Mathematica. We compared these adoption rates to 20 year compound annual growth rates for other technologies [19]. Mathematica adoption (CAGR = 3%) was comparable to broadcast capacity growth (6%) which is slowly evolving from analog to digital channels, while Python (40%) fell between telecom growth (28%), fueled by Internet and mobile phone adoption, and general-purpose computing growth (58%). Overall, programming language adoption rates were similar to recent U.S. government spending estimates for open source software (8%) [31]. Fortran is among the oldest languages and is, thus, not surprisingly more mature and experiencing slower growth. It is also perhaps not surprising that growth rates in open source scripting languages outpaced those of commercial development languages such as Visual Basic and Mathematica. However, using the Google Ngram Viewer and search, the frequency of JavaScript books and websites still exceeds those for Bioconductor. We also assessed search results from social networking, publication, employment, and intellectual property websites and generally found no correlation with results from other domains. The lack of correlation was surprising and may suggest that research publication patterns are further removed from industrial output than expected.

### Variable adoption of HIT

Medical informatics has been described as having a “long and delayed adolescence” which continues to “find itself in search of self-definition” [32], and this description could be applied to HIT as well. In 2010, Haux described the field as “relatively stable over the last 15 years” with “shifts during the last three years towards clinical order entry, natural language processing, formalization of guidelines, and the development of standards for patient records” [33]. More detailed historical timelines such as those available for programming languages would be valuable contributions to the HIT literature, but they are difficult to construct due to challenges of heterogeneity and nomenclature. Among HIT, HIS have a mixed history of successes as well as expensive and challenging implementations. For example, the HELP hospital information system has been operational since 1967. The system initially supported a heart catheterization laboratory and has since expanded to provide decision support for various functions [34]. On the other hand, many examples of failed implementations exist. Perhaps in contrast, the benefits of RS have been supported in several systematic reviews. Examples include reminders for adherence to tuberculosis clinic appointments [35]; reminders to improve preventive practices for vaccinations, cancer screening, and cardiovascular risk [36]; and reminders to improve immunization rates [37].

In our data, adoption of HIS was variable over time. The fraction of publications per year has grown most rapidly for LIS and most slowly for RIS. HIS have grown to have the greatest presence, perhaps due to demand, while RS were second, perhaps due to generalizability. Relative to the programming languages, growth in HIT publications were more modest. Using Google Ngram Viewer and search, the frequency of HIS books exceeds that of other HIT. Interestingly, the RIS adoption rate (CAGR = 4%) was similar to increases in biopsy procedures during the past decade performed by radiologists (8%) [38].

### Adoption rates and drivers

A seminal study by Coleman [39] examined the social processes of adoption of a new drug among 125 physicians in four cities over 15 months, from early adoption to widespread acceptance, and found differences in professional and, more importantly, social characteristics. The results suggest a snowball effect that may be applicable to popular technologies in the current study such as open source programming languages. However, that study examined one technology being adopted by various types of users, while in the current study, multiple technologies are being adopted by users who are not specifically characterized. A subsequent study by Kaluzny [40] discussed the societal drivers of healthcare innovation in detail, including the presence of public health, community service, and private practice entities and their organizational, technological, communication, temporal and social characteristics.

On a more theoretical level, a study by Granovetter [41] linked the “micro and macro levels” of social networks, arguing that the degree of overlap of networks varies directly with the strength of their ties to one another. The use of network analysis can thus be related to phenomena such as innovation diffusion and social mobility, organization, and cohesion. Analyses acknowledged as missing in that study, such as assessment of specialization and the developmental of network structure over time, are discussed in the current study.

### Minimal geographical influence

Even when innovations are successfully adopted in one location, they often disseminate slowly, if at all [42]. Borner et al. analyzed spatial diffusion patterns of information over 20 years among major U.S. research institutions [43]. Surprisingly, they found that the advent of the Internet did not increase the geographical distance over which information diffuses. As the number of published papers has increased, distance may become an impediment since authors were more likely to cite papers by authors at nearby institutions, suggesting that the “social component” of collaboration has become more important. Their analysis did not consider the subject matter of the publications. As opposed to allopatric or geographic speciation, when populations become isolated due to geographical barriers and undergo divergence due to different selective pressures, in sympatric speciation, speciation occurs in a population sharing the same geography. We see some evidence of both forces, suggesting a global technology community as well as local trends.

In this study, a world map shows countries of origin for publications and displays expected clusters in North America, Europe, East Asia, and Asia-Pacific. Within types of technology, there is considerable variation in the relative fractions of publication frequencies. There was little geographical correlation, further supporting the hypothesis that technology adoption is a virtual process.

### Languages form connected network components

George Box is credited with saying that all models are wrong, but some are useful. We chose a network approach to model technology adoption, though other strategies such as agent based models [44], social-cognitive theory [45], and the diffusion of innovation theory [46] have also been proposed. In previous network studies, Yousefi-Nooraie et al. and others have found an association between publication productivity and topological features [47]. As we observed in this study, they found that successful research centers showed denser, more cooperative networks.

Our network diagrams of studies as nodes and common authors as edges show the largest connected components represent modern scripting languages. Temporal network diagrams showed that nodes of similar time periods tend to be neighbors, though this is less frequently true among more mature languages. The two largest connected components among HIT represent HIS, and two of the next three biggest components represent LIS. Among programming languages, the largest connected component represents Bioconductor. The next largest, which surprisingly is also the only hybrid component, represents Perl and Python. Interestingly, based on a linear model, Bioconductor has more edges than expected and Perl has fewer, perhaps due to Bioconductor's high specialization and cooperativity and Perl's slower initial evolution. For Bioconductor and Perl, the largest connected components include well-defined topical clusters.

### Winning technologies are specialized, cooperative, competitive, and mutualistic

At the level of individual technologies, several ecological models can be useful to describe research collaboration: (1) we assessed specialization as the fraction of publications per technology occurring in a single journal; (2) we assessed mutualism and competition based on technologies published in similar journals; (3) we calculated cooperativity as the number of authors per publication; (4) and finally, for languages, we assessed evolution as the progression of time-to-event for new version releases.

More specialized IT may be prone to more rapid adoption. For example, early adopters of digital pathology were laboratories that needed to provide pathology services at great distances and needed technology to increase efficiency. The creation of standards for virtual slide pathology has facilitated adoption [48]. Because most of the programming languages in this study shared a common specialization niche--bioinformatics--we consider them to be potentially mutualistic, particularly among the scripting languages, or in competition, particularly the open source versus the commercial languages. On the other hand, the HIT have different and lower degrees of specialization. In terms of cooperativity, scripting languages and HIT were comparable. Older and commercial languages lagged.

Associations between other ecological strategies, such as cooperation and competition, and behaviors such as growth rate, the ability to survive in different environments, and the distribution of other species that co-inhabit an ecosystem have been observed across species. Appropriate strategies emerge: a niche with little co-habitation is associated with a slow growth rate, while ecological diversity with intense co-habitation can be associated with a faster rate [49]. Freilich et al. examined microorganism interactions in the literature to construct a network and demonstrate a pattern of association between species lifestyle and the number of co-occurring partners. They find relationships between resource competition--a research analogy might be funding--and growth rate [50]. Successful languages appear to have a mutualistic relationship--Python and Perl enabling each other--while waning languages appear to be the neutral party in commensal relationships or the losing party in a competitive relationship. It is unclear why, among languages, specialization and cooperativity were positively correlated, while among HIT they were negatively correlated. By time-to-event analysis, rates of language evolution were similar. Perl began as an exception, but around the release of version 5.8 rapidly accelerated its release schedule. Interestingly, Perl experienced rapid early adoption while its evolution in time-to-release was slower.

### Technology ecosystems are diverse and unstable

There are several biodiversity analogies relevant to technology: genetic diversity is like diversity within a language (e.g., indentation and commenting styles), species diversity is like differences among languages (e.g., strongly versus weakly typed), and ecological system diversity is like the degree of difference among a system of languages (e.g., interpreted versus compiled, open source versus commercial). Here, in two limited model “ecosystems”, we quantitatively examined species and system diversity. Ecological studies of food webs have reached inconsistent conclusions about the relationships between complexity or diversity with stability or persistence [51], [52]. Interestingly, as with specialization and cooperatively, we found divergent relationships between stability and biodiversity for HIT and languages. We found negative correlations for HIT, but among languages, we found positive relationships. For the two technology families, we calculated biodiversity using entropy, and we estimated stability as the correlation of publication numbers. Data indicate that diversity among languages grew in the first half of the decade and stabilized, while diversity among HIT has been variable but flat. It is perhaps a statistical artifact that biodiversity among languages appears to have stabilized. Estimates of short- and long-term stability found one-year correlation was positive while ten-year correlation was weakly negative, suggesting that the relative popularity of technologies is unstable, most likely due to rapidly changing, external technological and market drivers.

### Bridging publication and ecological systems

The range of IT methodologies, applications, and--more than ever--interactions in biomedical research and clinical practice has become extraordinarily complex. The cost and effort of implementing new technologies as well as the potential missed opportunities, in terms of scientific productivity and patient care, due to unsuccessful implementations demand that IT systems become better understood. Because peer-reviewed publications are a widely accepted and comprehensive permanent record of biomedical research, they present an invaluable resource for inquiry. However, as complex as IT and the research literature have become, traditional methods for analyzing and interpreting the data may no longer suffice. Networks are becoming increasingly appreciated for their ability to model complex social systems such as research co-authorship. Recently, network models have also been applied successfully to analyze ecological systems, but just as importantly, the behaviors of ecological systems have provided insights into the structure of network models. In the cases of both abstract networks and real world social and ecological systems, emergent behaviors arising from populations of individuals may be different than the simple sum of their parts.

We built this study on several key premises: first, that the biomedical literature could be used as a detailed and comprehensive description of technology adoption; second, that network models could be applied to understand the complex topology of IT publications; and third, that ecological models could further inform and enable interpretation of technology adoption and publication networks. There were many possible ways to conduct this analysis. We chose to model a publication as an organism in a population, where publications related to different types of technologies were analogous to organisms of different species. These publications, regardless of technology discussed, formed relationships based on shared authorship just as organisms might form relationships with other organisms of the same or different species. An implicit assumption is that publications and organisms are independent and can “take on a life of their own”. Groups of publications subsequently formed niches, often within the same or similar journals, just as groups of organisms might form a niche in a lake or a cave. These behaviors could be measured using abstract bibliometric network statistics such as co-authorship and density as well as ecological metrics such as specialization and cooperatively. As these niches combined to form “ecosystems”, in this case programming languages or HIT, emergent properties were observed related to system diversity and stability that are being studied with great interest in real-world deserts, oceans, and cities.

We constructed a mapping of technology, network, publication, and ecosystem entities (Table 1). We also included network and ecosystem metrics that could be applied to publications. Notably, there was a consistent ability to match features across all four domains, and most of the features were salient to the present analysis. Some results were surprising. The lack of correlation between publication counts and counts of downstream work products such as patents and books reinforces the notion that technology adoption and implementation are different phenomena. We were also able to make observations that link publication and ecosystem entities. For example, technologies with slower growth rates in terms of publication counts were associated with lower specialization in terms of journal of publication.

### Strengths, limitations, and future work

In this study, we performed quantitative analyses on a large set of biomedical publications spanning a decade with a focus on relevant biomedical information technologies. The study reproduced several previously reported relationships of publication and ecosystem networks. The study also extended these findings by unifying them, quantitatively and conceptually, by applying them to the dynamic problem of technology adoption. In particular, this study found that programming language and HIT growth was similar to reports of other technologies [19], but the lack of correlation between academic publication and downstream work products such as patents and books reinforces the notion that adoption and implementation are different phenomena [25]. Furthermore, HIT adoption was slower and more variable as has been described [32], though the benefits of RS [35]–[37] were reflected in faster growth. We also observed that HIT, a niche with little co-habitation or specialization, was associated with a slower growth rate, while intense co-habitation among languages was associated with a faster rate, as shown in nature [49]. In addition, we found divergent relationships between stability and biodiversity for HIT and languages [51], [52]. This study unified these various findings by quantifying and comparing technology adoption relationships using ecological and topological metrics. Thus, the analytical approaches and ecological models applied to IT research publications provide insight into species-level (e.g., individual languages) and system-level (e.g., languages v. HIT) characteristics.

There were also several implicit assumptions and associated limitations in this study. The programming languages and HIT were chosen based on perceived importance and lack of semantic ambiguity. Notable exclusions included the C and Java languages and HIT such as electronic medical records and decision support systems. No technology in this study experienced decreases in adoption. From ontological and implementation perspectives, programming languages and HIT are not, themselves, directly comparable. Additional studies would be needed to determine whether these results are reproduced in other classes of technologies and methodologies, such as databases, controlled vocabularies, computer hardware, and laboratory instruments. There are also well documented challenges around precision and recall of MEDLINE search terminology [53], [54]. Other limitations of the literature search include author name ambiguity, incomplete reporting of the Place field, and possible geographical publication biases. Web data from other domains introduce additional potential bias and confounding, and their comparison was not even possible for HIT due to the low number of results. Network analysis and comparability were influenced by sample size. Finally, ecological metrics were associative rather than causative.

Future work will be needed to assess the role of the complex forces in today's technological society that could affect adoption of programming languages and HIT. In ecology, an indicator species is one that is affected early by external trends [55], and such an awareness of extrinsic factors will be useful to interpret the trends in this study. For example, it is possible that web developers are moving from HTML to JavaScript in response to the demand for mobile applications. Other external forces include growth in security issues and cloud computing [23]. Ongoing analysis will be needed to assess these effects.

### Conclusion

In this study, we performed statistical analyses and constructed ecological models of information technology adoption based on the biomedical literature. We focused on programming languages used in bioinformatics research and health IT systems implemented in clinical settings. Adoption of HIT has been variable, while scripting languages have experienced rapid adoption and have grown to form large connected network components. We found that spatiotemporal relationships can facilitate adoption but are not sufficient. Dense, mutualistic “ecosystems” are associated with faster technology adoption. We propose that a better understanding of which technologies get adopted, as well as how and why, can potentially facilitate their design and distribution as well as lead to socioeconomic benefits such as intellectual property production and employment opportunities.

## Supporting Information

Figure S1 **Culturomics trends.** We compared frequency trends for publications of technologies with Google Labs' Ngram Viewer. We observed some divergence from the scientific literature. For example, (A) among HIT, books on electronic medical records (not included in this study) have increased since the mid-1990s, but (B) the frequency of HIS books exceeds that of other types. Among programming languages, (C) the number of Perl books spiked and fell dramatically in the early part of the decade, but (D) the frequency of JavaScript books still exceeds those for Bioconductor.
(PPT)

Figure S2 **Plot of search results in Nature Jobs versus numbers of publications for programming languages.** The unadjusted p-value is significant and suggests a relationship between research output and socioeconomic benefit, but it may be the result of multiple hypothesis testing.
(PPT)

Figure S3 **Geographic variation.** (A) Within technology families (languages shown here), there is considerable geographical variation in the relative publication frequencies. (B) Among programming languages, England publishes more frequently on scripting languages, while the US publishes more frequently using commercial products. Specifically, 75% of Bioconductor publications and 66% of Perl publications originated in England, while 46% of Visual Basic publications and 47% of Mathematica publications originated in the United States. The two countries were nearly equal in JavaScript (44% versus 42%) and Fortran (40% versus 43%) publications.
(PPT)

Figure S4 **Color-coded countries according to the frequency of published technologies.** Panels depict (A) RS, (B) RIS, (C) LIS, (D) HIS. Countries shown in red indicate a frequency of greater than 60%, orange indicates 50–60%, yellow indicates 40–50%, green indicates 30–40%, cyan indicates 20–30%, blue indicates 10–20%, and purple indicates less than 10%.
(PPT)

Figure S5 **Summary of common journals of publication suggest different secondary specializations.** Python is commonly used in neuroinformatics studies, Mathematica in engineering and imaging, JavaScript in medical informatics, and Visual Basic in behavioral research.
(PPT)

Figure S6 **Publication stability.** To explore whether past publication frequencies describe stability and predict future frequencies, we calculated a Pearson correlation matrix for each technology across the ten years from 2001 to 2010. We found that while publication frequencies were correlated with frequencies from previous years (r>0.80), the correlation with data from three years earlier was variable, suggesting that the relative popularity of technologies is unstable and that current states are not predictive of future states, even within a short year time horizon.
(PPT)
