# Supplementary figures and images for: Informatics Technology Mimics Ecology: Dense, Mutualistic Collaboration Networks Are Associated with Higher Publication Rates
Source: PLoS One. 2012 Jan 18;7(1):e30463. doi: 10.1371/journal.pone.0030463 (PMC3261203; doi:10.1371/journal.pone.0030463)

## Slide 1
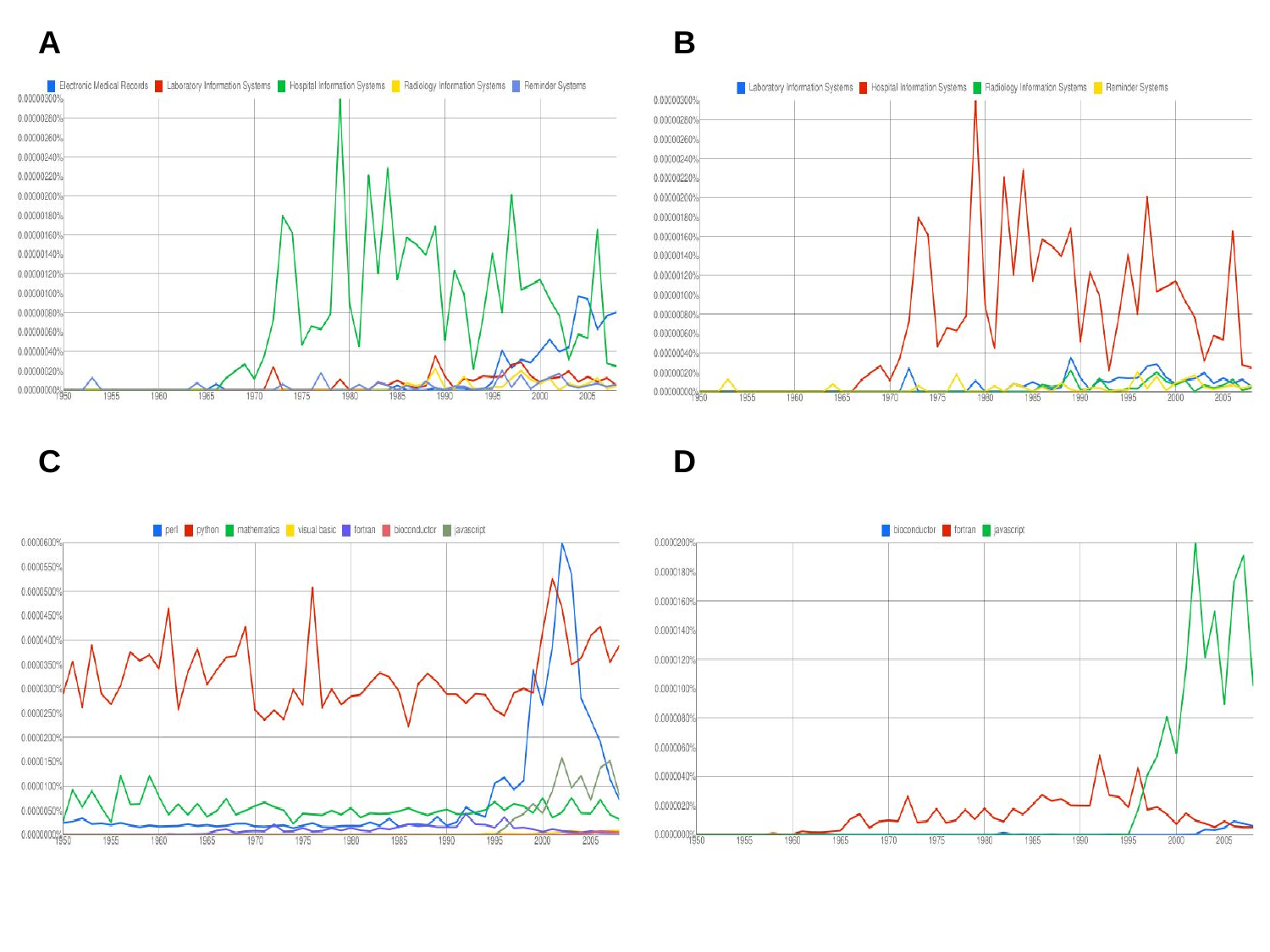

A					B
C					D

Supplement: Figure S1 — Culturomics trends. We compared frequency trends for publications of technologies with Google Labs' Ngram Viewer. We observed some divergence from the scientific literature. For example, (A) among HIT, books on electronic medical records (not included in this study) have increased since the mid-1990s, but (B) the frequency of HIS books exceeds that of other types. Among programming languages, (C) the number of Perl books spiked and fell dramatically in the early part of the decade, but (D) the frequency of JavaScript books still exceeds those for Bioconductor. (PPT) [file pone.0030463.s001.ppt]

## Slide 1
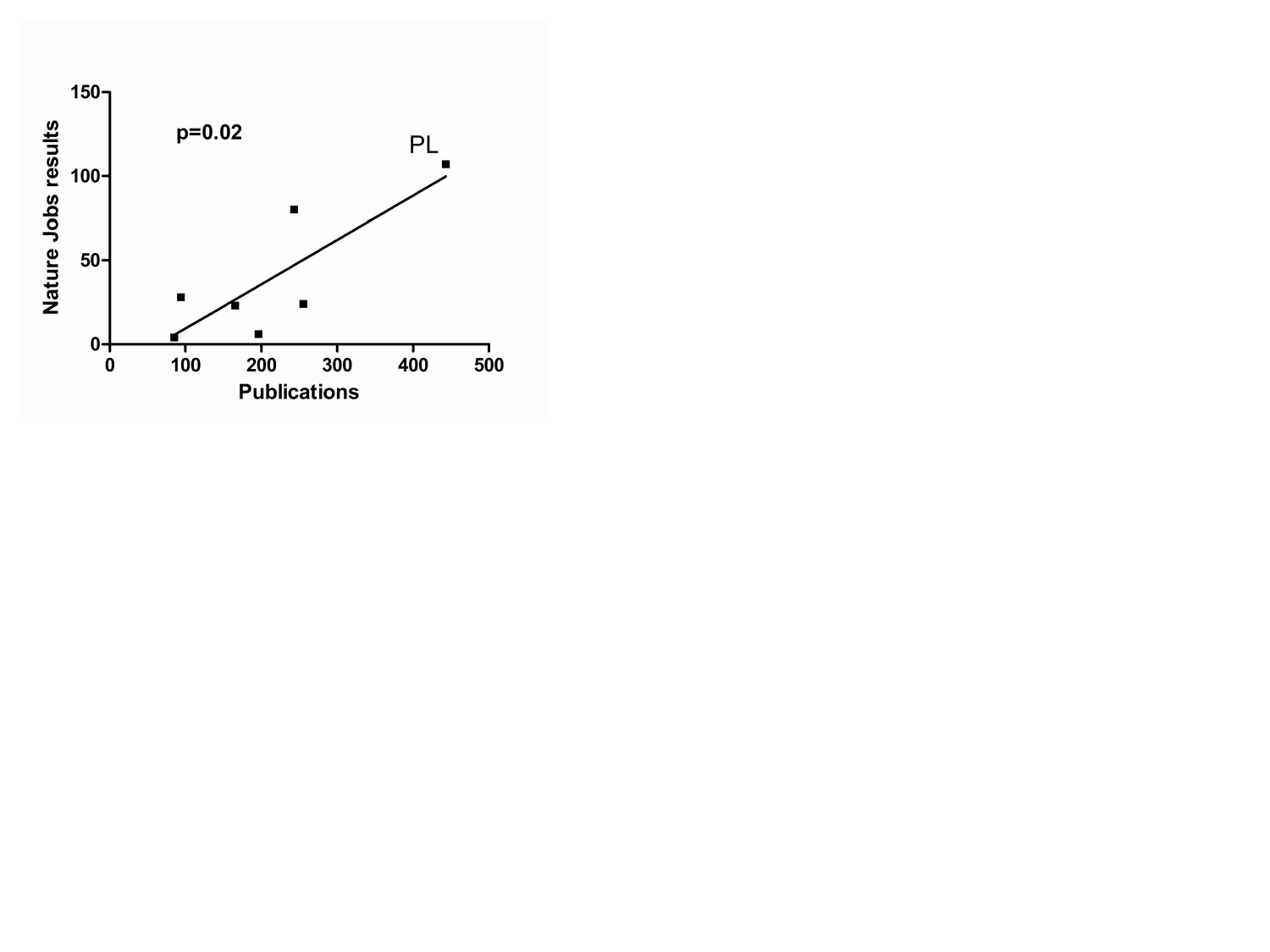

Supplement: Figure S2 — Plot of search results in Nature Jobs versus numbers of publications for programming languages. The unadjusted p-value is significant and suggests a relationship between research output and socioeconomic benefit, but it may be the result of multiple hypothesis testing. (PPT) [file pone.0030463.s002.ppt]

## Slide 1
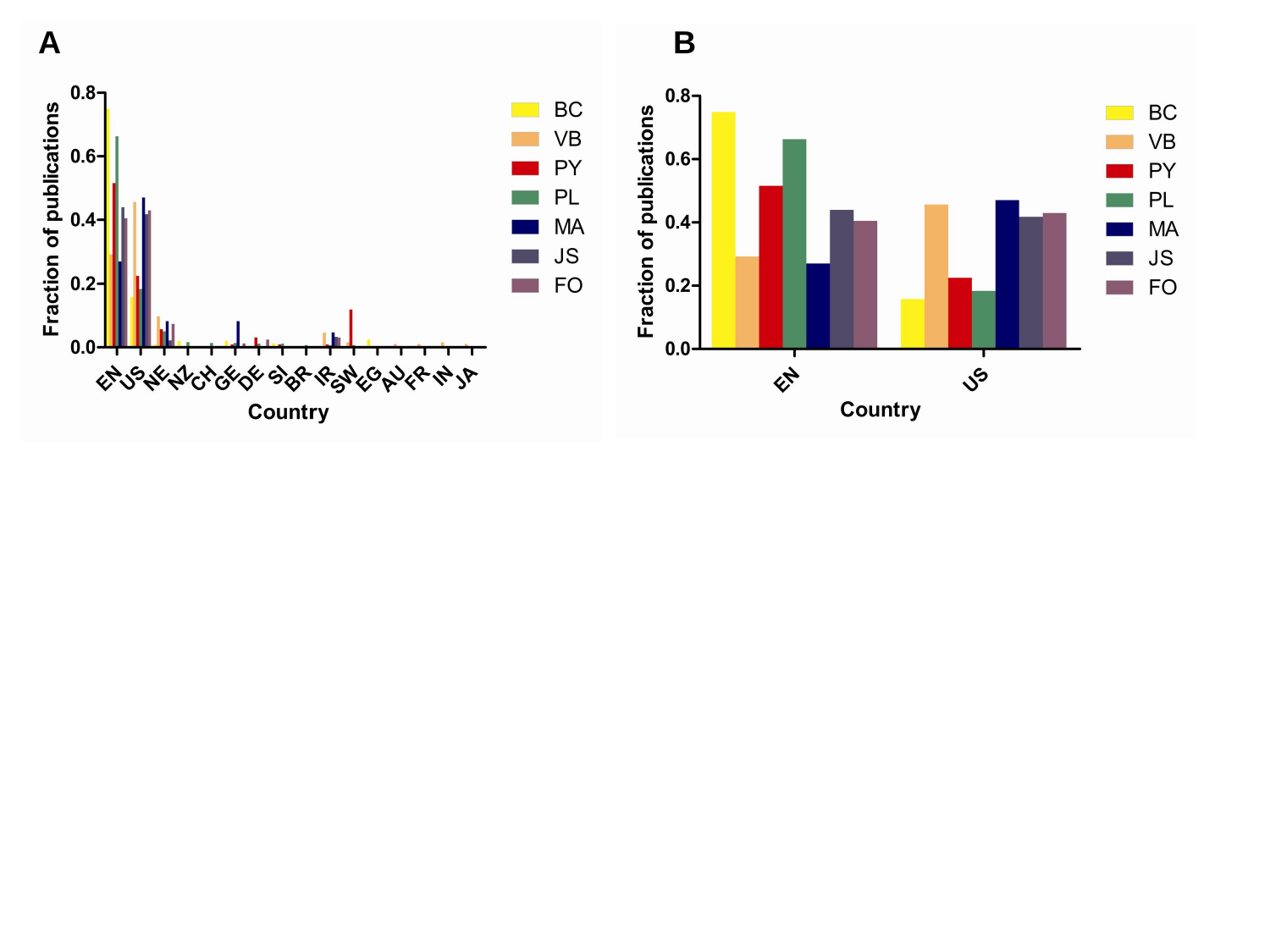

A					B

Supplement: Figure S3 — Geographic variation. (A) Within technology families (languages shown here), there is considerable geographical variation in the relative publication frequencies. (B) Among programming languages, England publishes more frequently on scripting languages, while the US publishes more frequently using commercial products. Specifically, 75% of Bioconductor publications and 66% of Perl publications originated in England, while 46% of Visual Basic publications and 47% of Mathematica publications originated in the United States. The two countries were nearly equal in JavaScript (44% versus 42%) and Fortran (40% versus 43%) publications. (PPT) [file pone.0030463.s003.ppt]

## Slide 1
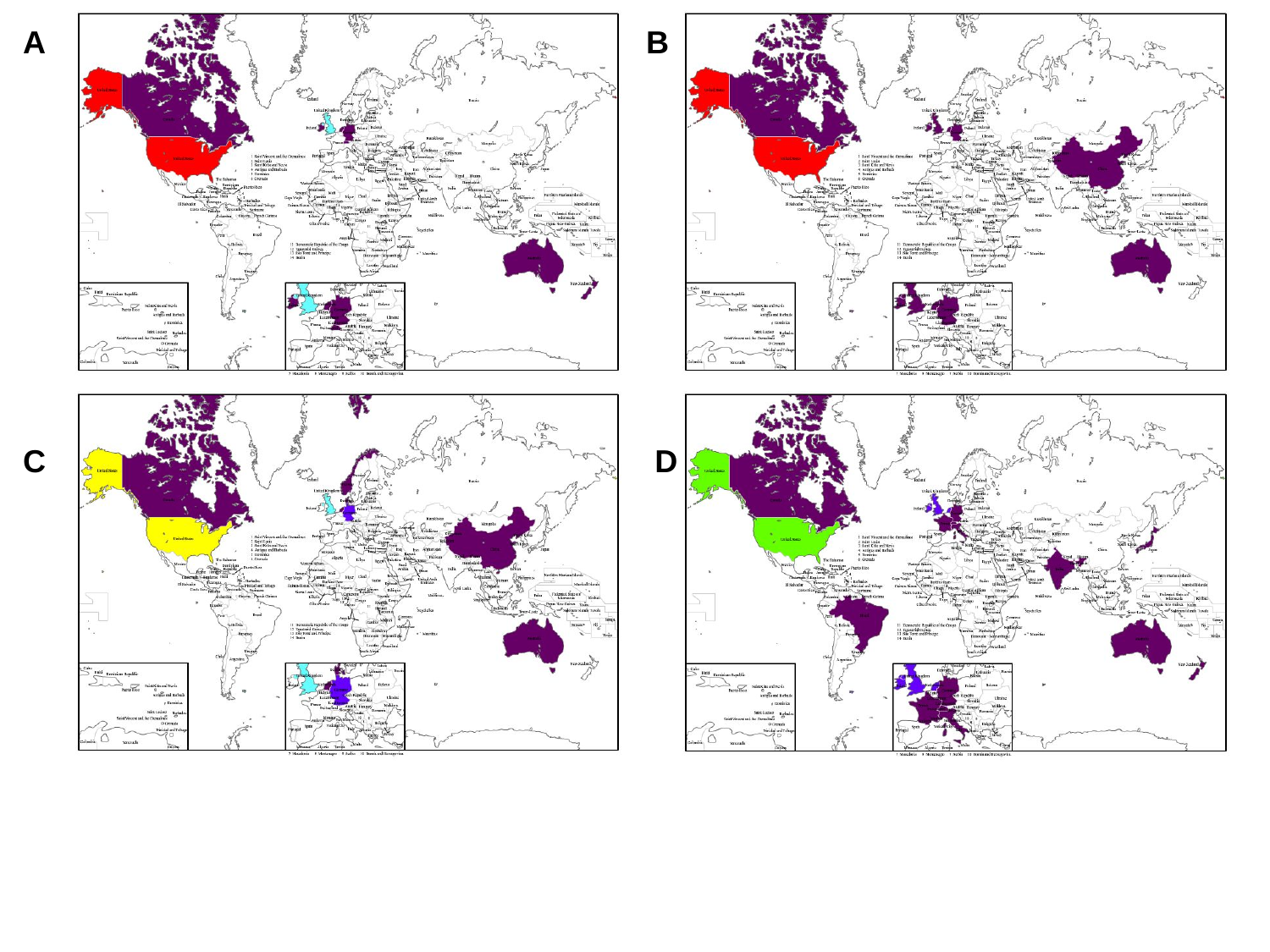

A				 B
C				 D

Supplement: Figure S4 — Color-coded countries according to the frequency of published technologies. Panels depict (A) RS, (B) RIS, (C) LIS, (D) HIS. Countries shown in red indicate a frequency of greater than 60%, orange indicates 50–60%, yellow indicates 40–50%, green indicates 30–40%, cyan indicates 20–30%, blue indicates 10–20%, and purple indicates less than 10%. (PPT) [file pone.0030463.s004.ppt]

## Slide 1
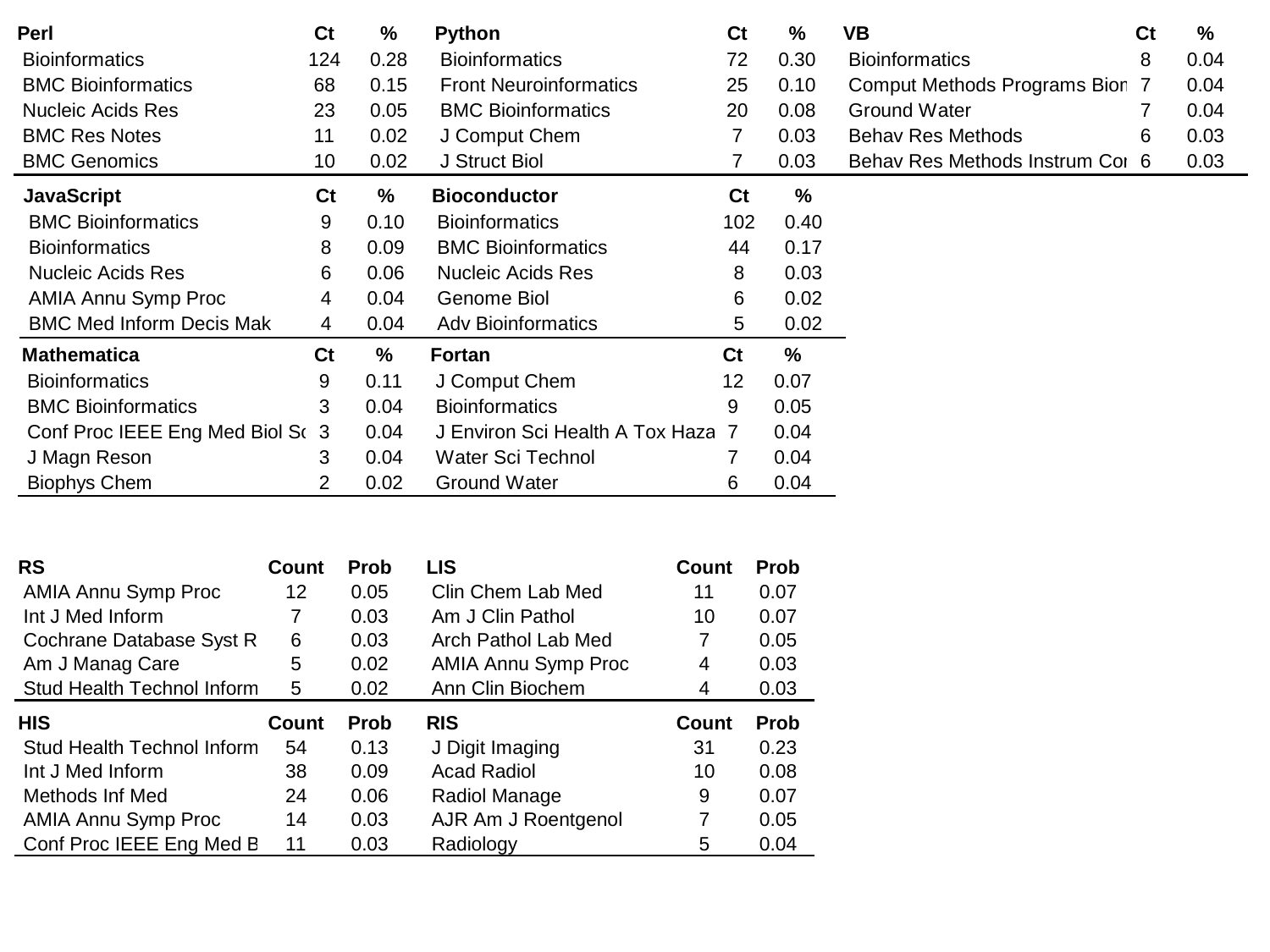

Supplement: Figure S5 — Summary of common journals of publication suggest different secondary specializations. Python is commonly used in neuroinformatics studies, Mathematica in engineering and imaging, JavaScript in medical informatics, and Visual Basic in behavioral research. (PPT) [file pone.0030463.s005.ppt]

## Slide 1
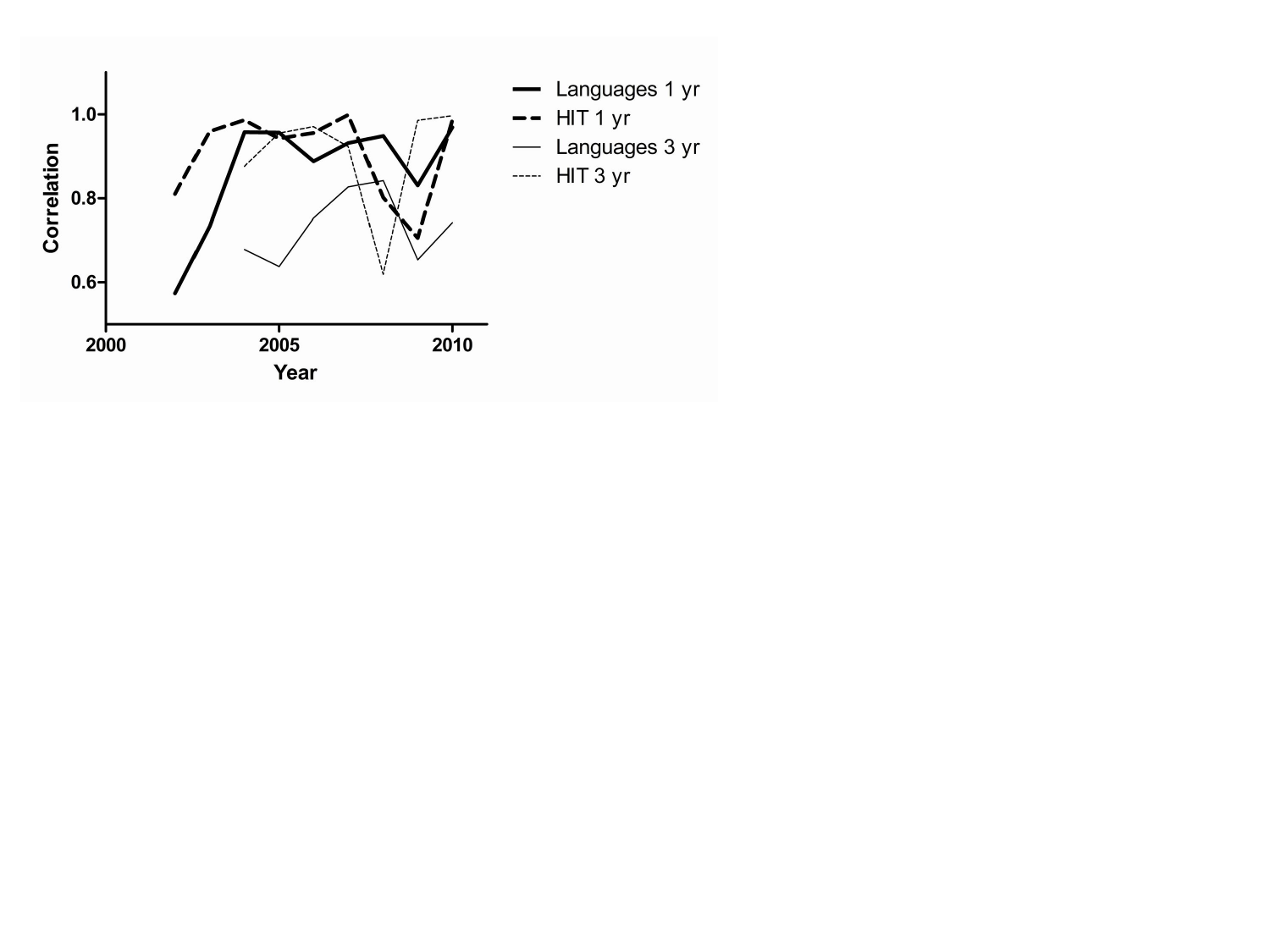

Supplement: Figure S6 — Publication stability. To explore whether past publication frequencies describe stability and predict future frequencies, we calculated a Pearson correlation matrix for each technology across the ten years from 2001 to 2010. We found that while publication frequencies were correlated with frequencies from previous years (r>0.80), the correlation with data from three years earlier was variable, suggesting that the relative popularity of technologies is unstable and that current states are not predictive of future states, even within a short year time horizon. (PPT) [file pone.0030463.s006.ppt]
